# The overall fractions of coronary heart diseases and depression attributable to multiple dependent psychosocial work factors in Europe

**DOI:** 10.1007/s00420-024-02067-x

**Published:** 2024-04-15

**Authors:** Isabelle Niedhammer, Hélène Sultan-Taïeb, Jean-François Chastang

**Affiliations:** 1 INSERM, Univ Angers, Univ Rennes, EHESP, Irset (Institut de recherche en santé, environnement et travail) - UMR_S 1085, Epidemiology in Occupational Health and Ergonomics (ESTER) Team, Angers, France; 2 School of Management, Université du Québec à Montréal (ESG-UQAM), Montréal, Canada

**Keywords:** Attributable fraction, Cardiovascular diseases, Mental health, Job strain, Effort-reward imbalance, Job insecurity, Long working hours, Workplace bullying

## Abstract

**Objectives:**

The literature is nonexistent on the assessment of overall fractions of diseases attributable to multiple dependent psychosocial work factors. The objectives of the study were to calculate the overall fractions of coronary heart diseases (CHD) and depression attributable to multiple dependent psychosocial work factors in 35 European countries.

**Methods:**

We used already published fractions of CHD and depression attributable to each of the following psychosocial work factors: job strain, effort-reward imbalance, job insecurity, long working hours, and workplace bullying. We took all exposures and their correlations into account to calculate overall attributable fractions. Wald tests were performed to test differences in these overall attributable fractions between genders and between countries.

**Results:**

The overall fractions of CHD and depression attributable to all studied psychosocial work factors together were found to be 8.1% [95% CI: 2.0-13.9] and 26.3% [95% CI: 16.2–35.5] respectively in the 35 European countries. There was no difference between genders and between countries.

**Conclusion:**

Our study showed that the overall fractions attributable to all studied psychosocial work factors were substantial especially for depression. These overall attributable fractions may be particularly useful to evaluate the burden and costs attributable to psychosocial work factors, and also to inform policies makers at European level.

## Introduction

Psychosocial work factors were found to be associated with various health outcomes and especially with mental health and cardiovascular outcomes (Niedhammer et al. 2021). Although the etiological effects of these factors have been explored extensively, the fractions of diseases attributable to these factors remain less frequently studied and evaluated. One of the reasons for this may be that causality is still under debate (Mikkelsen et al. 2021; Rugulies et al. 2023). These attributable fractions (AFs) can be seen as important tools to estimate the burden in terms of morbidity and mortality attributable to psychosocial work factors and are necessary for the calculation of the costs of diseases attributable to these factors (Sultan-Taïeb et al. 2013, 2022).

Previous studies assessing the fractions of diseases attributable to psychosocial work factors were seldom in the literature. These studies mostly focused on a single exposure, job strain, i.e. the combination of high psychological demands and low decision latitude, as defined by Karasek (Karasek et al. 1998), and provided estimates for the fractions of mental health outcomes (Lamontagne et al. 2008; Nurminen and Karjalainen 2001) or cardiovascular diseases (Kivimaki et al. 2012) attributable to job strain. In one of our earlier publications (Niedhammer et al. 2022), we calculated the fractions of coronary heart diseases (CHD) and depression attributable to five different psychosocial work factors separately in 35 European countries in 2015. These factors were job strain, effort-reward imbalance (ERI) (i.e. the imbalance between high effort and low reward), job insecurity, long working hours, and workplace bullying, considered as crucial exposures from the psychosocial work environment.

However, the literature is nonexistent for the calculation of the overall fractions of diseases attributable to multiple psychosocial work factors. Indeed, whereas the calculation of AF for a single exposure relies on both the prevalence of exposure and the relative risk (RR) associated with the exposure, the calculation of overall AF for multiple exposures requires the prevalence of exposure for the different combinations of exposures and the RRs associated with these combinations, which were not available in the literature. Consequently, in our earlier publication (Niedhammer et al. 2022), we provided minimum and maximum values for the overall AFs, but not single estimates. The minimum value corresponded to the hypothesis of total dependence between exposures. The maximum value corresponded to the hypothesis of independence between exposures. These extreme hypotheses are in general not validated, as psychosocial work factors are neither totally dependent, nor totally independent, but in between.

The objective of the study was to calculate the overall fractions of CHD and depression attributable to multiple dependent psychosocial work factors, taking the correlations between exposures into account, in Europe.

## Methods

In our earlier publication (Niedhammer et al. 2022), we calculated the weighted prevalence of each exposure (job strain, ERI, job insecurity, long working hours, and workplace bullying) using the data from the 2015 European working conditions survey (EWCS from EUROFOUND, sample of 35,571 employees) for 35 European countries and each country separately. RR estimates for the associations of each exposure with CHD and depression were extracted from literature reviews and meta-analyses (Niedhammer et al. 2021). Only the RR estimate between workplace bullying and CHD was missing in the literature. The RRs were obtained from prospective studies and were adjusted for gender, age, and socioeconomic status, or the closest adjustment. We were then able to calculate the fractions of CHD and depression attributable to each exposure using both the prevalence of exposure and RR.

In the present study, we used these AFs for each exposure to calculate the overall fractions of CHD and depression attributable to all studied exposures together, using a formula that provides an approximate value of the overall AF while taking the correlations between exposures into account (Niedhammer and Chastang 2021). This formula was developed on the basis of Miettinen’s formula (Miettinen 1974) modified to take the tetrachoric correlations between exposures into account, and more precisely the mean correlation of each exposure with the other exposures. Indeed, Miettinen’s formula assumes the independence between exposures, which was an assumption that was not validated. Miettinen’s formula was thus modified as follows:

1 $$Overall\,AF\, = \,1\, - \,\prod\limits_{i = 1}^p {\,\left[ {1\, - \,AF\,\left( i \right)} \right]} \,Miettine{n^\prime }s\,formula$$

with p the number of exposures, under the assumption of independence between exposures

2 $$\eqalign{ Approximate\,overall\,AF\, & = \,1\, - \,\prod\limits_{i = 1}^p {\left[ {1 - v\,\left( i \right)\, \times \,AF\,\left( i \right)} \right]} \cr & Niedhammer\,{\rm{\& }}\,Chastan{g^\prime }s\,formula \cr}$$

with *v(i)* the weight for exposure *i* calculated from:

$$1-mean\, tetrachoric \, correlation \, coefficient\,\left(i\right)$$

The differences in the overall AFs between genders and between countries were tested using the Wald test. Statistical analyses were performed using SAS software.

## Results

The mean tetrachoric correlations between each exposure and the other exposures ranged between 0.14 and 0.50. The highest mean correlation was found for ERI with the other exposures, and the lowest mean correlation was found for long working hours with the other exposures.

The results for the overall AFs are presented in Table 1 for CHD and Table 2 for depression. The results showed that the overall fraction of CHD attributable to the four psychosocial work factors of job strain, ERI, job insecurity, and long working hours was 8.1% [95% CI: 2.0-13.9] for all 35 European countries (Table 1). No differences between genders and between countries were observed. The overall fraction of depression attributable to the five psychosocial work factors of job strain, ERI, job insecurity, long working hours, and workplace bullying was 26.3% [95% CI: 16.2–35.5] for all 35 European countries (Table 2). No differences between genders and between countries were found.

**Table 1:** Overall fractions of coronary heart diseases attributable to four psychosocial work factors (job strain, effort-reward imbalance, job insecurity, long working hours) in Europe in 2015

%	All		Men		Women	
	AF	95% CI	AF	95% CI	AF	95% CI
Albania	11.9	[2.8–20.3]	12.4	[2.6–21.4]	11.4	[2.6–19.7]
Austria	6.0	[1.3–10.9]	6.4	[1.2–11.4]	5.7	[1.1–10.1]
Belgium	6.7	[1.6–11.6]	7.3	[1.7–12.7]	6.1	[1.4–10.7]
Bulgaria	6.4	[1.4–11.3]	7.2	[1.3–12.8]	5.7	[1.1–10.8]
Croatia	10.0	[2.4–17.1]	10.6	[2.4–18.4]	9.3	[2.2–16.2]
Cyprus	9.4	[2.4–16.1]	9.7	[2.3–16.8]	9.1	[2.8–15.6]
Czech Rep	8.1	[1.9–14.1]	8.3	[1.7–14.6]	7.9	[1.7–13.9]
Denmark	5.6	[1.2–9.9]	4.9	[0.9–8.9]	6.4	[1.3–11.2]
Estonia	7.6	[1.8–13.1]	8.5	[1.8–14.9]	6.7	[1.4–11.8]
Finland	6.2	[1.4–11.0]	5.6	[1.0–10.0]	6.8	[1.4–12.0]
France	7.3	[1.7–12.6]	7.0	[1.5–12.4]	7.5	[1.7–13.0]
FYROM	9.1	[2.1–15.8]	10.3	[2.3–17.8]	7.7	[1.5–13.6]
Germany	5.5	[1.3–9.6]	5.8	[1.3–10.1]	5.3	[1.2–9.3]
Greece	12.4	[3.2–21.0]	12.0	[2.8–20.6]	12.8	[3.2–21.8]
Hungary	8.3	[2.0-14.4]	9.0	[1.9–15.6]	7.8	[1.7–13.6]
Ireland	7.7	[1.7–13.4]	8.4	[1.6–14.8]	7.0	[1.4–12.4]
Italy	8.4	[2.1–14.5]	8.6	[1.9–14.9]	8.3	[1.9–14.4]
Latvia	7.3	[1.7–12.7]	8.2	[1.7–14.4]	6.5	[1.4–11.6]
Lithuania	7.1	[1.7–12.4]	6.9	[1.3–12.4]	7.3	[1.6–12.7]
Luxembourg	5.9	[1.3–10.4]	5.2	[0.8–9.5]	6.7	[1.4–11.8]
Malta	5.1	[1.0-9.2]	6.4	[1.1–11.5]	3.4	[0.4–6.4]
Montenegro	10.8	[2.4–18.6]	10.8	[2.2–18.9]	10.7	[2.3–18.7]
Netherlands	8.3	[2.0-14.4]	8.4	[1.8–14.8]	8.2	[1.9–14.3]
Norway	4.6	[1.0-8.1]	4.8	[0.8–8.7]	4.3	[0.8–7.7]
Poland	9.4	[2.3–16.1]	10.7	[2.5–18.5]	8.1	[1.9–14.1]
Portugal	8.5	[2.0-14.7]	9.5	[1.9–16.8]	7.6	[1.6–13.3]
Romania	8.9	[2.1–15.3]	9.0	[1.9–15.7]	8.7	[2.0-15.2]
Serbia	10.9	[2.6–18.8]	11.8	[2.6–20.4]	10.1	[2.2–17.5]
Slovakia	6.5	[1.4–11.4]	6.6	[1.1–11.8]	6.4	[1.3–11.4]
Slovenia	10.3	[2.6–17.6]	10.6	[2.6–18.3]	10.0	[2.5–17.2]
Spain	10.9	[2.9–18.5]	11.2	[2.9–19.0]	10.7	[2.8–18.1]
Sweden	6.4	[1.4–11.2]	6.9	[1.4–12.3]	5.8	[1.2–10.4]
Switzerland	6.1	[1.4–10.7]	6.5	[1.3–11.5]	5.7	[1.1–10.2]
Turkey	11.6	[2.7–20.0]	12.1	[2.8–20.8]	10.5	[2.2–18.2]
UK	7.6	[1.8–13.1]	8.1	[1.8–14.1]	7.0	[1.5–12.3]
35 countries	8.1	[2.0-13.9]	8.5	[2.1–14.6]	7.7	[2.0-13.2]
p-value	NS		NS		NS	

AF: attributable fraction

All AFs were significantly different from 0

FYROM: Former Yugoslav Republic of Macedonia

p-value for the comparison between countries; NS: not significant

**Table 2:** Overall fractions of depression attributable to five psychosocial work factors (job strain, effort-reward imbalance, job insecurity, long working hours, workplace bullying) in Europe in 2015

%	All		Men		Women	
	AF	95% CI	AF	95% CI	AF	95% CI
Albania	29.2	[16.8–40.3]	30.2	[15.9–42.6]	28.8	[16.3–40.0]
Austria	23.9	[13.9–33.0]	23.7	[12.4–33.9]	24.0	[13.4–33.6]
Belgium	26.4	[16.1–35.7]	26.6	[15.7–36.5]	26.0	[15.6–35.5]
Bulgaria	17.5	[9.3–25.1]	18.4	[8.8–27.4]	16.8	[8.8–24.4]
Croatia	29.0	[17.0-39.7]	30.3	[16.6–42.2]	27.6	[15.5–38.4]
Cyprus	28.8	[17.6–39.0]	29.3	[17.1–40.2]	28.4	[16.4–39.1]
Czech Rep	23.4	[12.7–33.8]	23.8	[11.6–34.6]	23.1	[11.6–33.4]
Denmark	20.2	[11.2–28.6]	17.3	[8.2–25.8]	23.2	[12.4–32.9]
Estonia	22.6	[12.8–32.0]	25.2	[12.6–36.4]	20.1	[9.8–29.5]
Finland	22.3	[12.3–31.5]	17.5	[7.7–26.6]	26.2	[14.4–36.7]
France	31.8	[20.3–42.0]	30.0	[18.2–40.5]	33.4	[21.3–44.1]
FYROM	28.2	[16.1–39.0]	31.7	[17.5–44.0]	23.6	[12.1–33.9]
Germany	22.0	[13.1–30.8]	22.4	[12.8–31.2]	21.5	[12.3–30.0]
Greece	33.5	[20.4–45.0]	32.2	[18.4–44.2]	35.0	[20.7–47.4]
Hungary	24.0	[13.2–33.7]	24.4	[13.5–34.4]	23.6	[12.0-34.2]
Ireland	30.2	[18.1–41.0]	30.7	[16.6–43.0]	29.4	[16.7–40.6]
Italy	24.5	[13.8–34.2]	25.2	[13.2–35.9]	23.8	[12.6–33.9]
Latvia	22.9	[12.5–32.4]	23.8	[11.8–34.6]	22.1	[11.3–31.8]
Lithuania	24.7	[14.8–34.3]	23.5	[11.6–34.3]	25.6	[14.3–35.9]
Luxembourg	26.8	[16.2–36.5]	23.3	[12.3–33.3]	30.3	[18.2–41.1]
Malta	20.8	[11.4–29.4]	24.2	[12.8–34.5]	16.0	[7.2–24.2]
Montenegro	29.9	[17.1–41.2]	30.3	[16.6–42.2]	29.4	[15.3–41.7]
Netherlands	28.0	[16.2–38.6]	27.6	[14.6–39.1]	28.4	[15.7–39.6]
Norway	18.0	[9.8–25.7]	18.5	[8.9–27.3]	17.6	[8.9–25.6]
Poland	24.3	[13.5–34.2]	26.9	[14.6–38.0]	21.8	[11.2–31.6]
Portugal	22.9	[12.5–32.5]	24.9	[12.6–35.9]	21.2	[10.6–30.9]
Romania	28.2	[16.5–38.7]	26.9	[14.2–38.2]	29.6	[16.6–41.0]
Serbia	31.4	[18.2–42.8]	32.1	[17.4–44.8]	30.6	[16.8–42.5]
Slovakia	21.5	[11.9–30.3]	20.2	[9.8–29.8]	22.5	[11.8–32.4]
Slovenia	31.0	[18.7–41.9]	30.4	[17.4–41.9]	31.6	[18.6–42.9]
Spain	31.1	[19.1–41.7]	31.5	[19.0-42.5]	30.6	[18.5–41.4]
Sweden	21.8	[12.0-30.7]	21.3	[10.3–31.2]	22.2	[11.8–31.7]
Switzerland	22.0	[12.3–30.9]	22.0	[11.4–31.6]	22.0	[11.3–31.8]
Turkey	30.0	[17.6–41.0]	30.6	[17.5–42.0]	28.5	[15.8–39.7]
UK	26.4	[15.8–35.9]	26.9	[15.5–37.1]	25.8	[14.8–35.7]
35 countries	26.3	[16.2–35.5]	26.6	[16.2–35.9]	26.0	[16.1–35.0]
p-value	NS		NS		NS	

AF: attributable fraction

All AFs were significantly different from 0

FYROM: Former Yugoslav Republic of Macedonia

p-value for the comparison between countries; NS: not significant

## Discussion

The study showed that the overall fractions of CHD and depression attributable to all studied psychosocial work factors together were 8.1% [95% CI: 2.0-13.9] and 26.3% [95% CI: 16.2–35.5] respectively in the 35 European countries. There was no difference between genders and between countries.

The comparison with the literature was not possible as there was no previous study evaluating the overall fractions of cardiovascular diseases or mental disorders attributable to multiple psychosocial work factors.

As already mentioned in the strengths of our earlier study (Niedhammer et al. 2022), we studied various psychosocial work factors including well-known concepts, and both cardiovascular and mental health outcomes. We used a large study sample from a European survey (EWCS), that covered all European countries, to estimate the prevalence of exposure, and literature reviews and meta-analyses for the estimates of RR, in order to calculate AFs for each exposure. We were then able in the present study to calculate estimates for overall AFs for both genders and for Europe as a whole (35 countries) and for each country. We tested the differences in these overall AFs between genders and between countries. However, although differences in the AFs for each exposure were found between genders and between countries, no difference was found in the overall AFs. This might be explained by differences between genders (respectively, between countries) that were not the same according to the studied exposure, leading to reduced and even suppressed differences between genders (respectively, between countries) for the overall AFs. The major strength of the study was the calculation of these overall AFs that allowed to take all studied exposures and their correlations into account, making our study the first one in the topic of psychosocial work factors.

There were however some limitations to our study. The main psychosocial work factors, recognized as risk factors for the studied outcomes, were explored, but some others may be missing such as organisational injustice, consequently, our estimates for overall AFs should not be considered as overall fractions attributable to all psychosocial work factors. The measurement of psychosocial work factors using the EWCS data was based on proxies as validated questionnaires were not available. This may have led to a lack of precision and misclassification. Furthermore, there may be slight differences in the definition of the exposures studied in the EWCS data and those studied in the published studies from which RRs were calculated using meta-analysis in the reviews. There may also be slight differences between these published studies (see Supplementary Appendix in our previous study) (Niedhammer et al. 2022). As the comparison between countries was based on the Wald test (i.e. comparison with the mean value for all countries), our study may underestimate differences between countries. A further limitation was the use of the formula for the calculation of the overall AF (Niedhammer and Chastang 2021) that does not provide an exact value but an approximate value, as the literature did not provide the RRs associated with combined exposures. However, we showed that this approximation was very satisfactory previously (Niedhammer and Chastang 2021). In the absence of RRs for all combinations of exposures, our method may be the only approach we could use to date. Only very few studies provided estimates of RRs for combinations of 2 or 3 exposures in the literature (Dragano et al. 2017; Juvani et al. 2018; Lavigne-Robichaud et al. 2023) and reported that RRs for combined exposures may be higher than RRs for exposures separately, suggesting a potential underestimation of our estimates for overall AFs. On the other hand, our results for overall AFs may be overestimated because of a publication bias. Indeed, to calculate the AFs, we used RRs derived from meta-analysis of various literature reviews based on published studies. We cannot exclude that these RRs may be overestimated as unpublished studies, that may be more likely to report non-significant results, were not taken into account.

Our findings underlined that the overall fractions of CHD and depression attributable to all studied psychosocial work factors were substantial, especially for depression. Our study may be the first one to provide estimates for the overall fractions of CHD and depression attributable to multiple psychosocial work factors at European level. Such overall AFs can allow to estimate the overall burden in terms of morbidity and mortality, as well as the overall costs in monetary units of disease attributable to multiple dependent exposures, without double counting. As psychosocial work factors are modifiable risk factors, these findings may be particularly useful at European level to contribute to the definition of regulations and preventive strategies oriented towards the psychosocial work environment.

## Data Availability

The data that support the findings of this study are not openly available due to reasons of sensitivity and are available from the corresponding author upon reasonable request.

## Declarations

### Disclaimer

The sole responsibility for the content of this article lies with the authors. The European Commission or European Trade Union Institute (ETUI) are not responsible for any use that may be made of the information contained therein.
